# Synthesis of Iron Oxides and Influence on Final Sizes and Distribution in Bacterial Cellulose Applications

**DOI:** 10.3390/polym15153284

**Published:** 2023-08-03

**Authors:** Thaís Cavalcante de Souza, Andréa Fernanda de Santana Costa, Gloria Maria Vinhas, Leonie Asfora Sarubbo

**Affiliations:** 1 Center of Exact and Natural Sciences, Department of Materiais Science, Federal University of Pernambuco (UFPE), Rua Professor Moraes Rêgo, n. 1235, Cidade Universitária, Recife 50670-901, Brazil; thsouza221@gmail.com (T.C.d.S.); gloria.vinhas@ufpe.br (G.M.V.); 2 Advanced Institute of Technology and Innovation (IATI), Rua Potyra, n. 31, Prado, Recife 50751-310, Brazil; andrea.santana@ufpe.br; 3 Communication and Design Center, Centro Acadêmico da Região Agreste, Federal University of Pernambuco (UFPE), BR 104, Km 59, s/n, Nova Caruaru, Caruaru 50670-901, Brazil; 4 Department of Chemical Engineering, Federal University of Pernambuco (UFPE), Avenida dos Economistas—Cidade Universitária, Recife 50740-590, Brazil; 5 UNCAP Icam Tech School, Catholic University of Pernambuco (UNICAP), Rua do Príncipe, n. 526, Boa Vista, Recife 50050-900, Brazil

**Keywords:** iron oxides, synthesis, SPIONs, magnetism, bacterial cellulose, new materials

## Abstract

Iron oxide nanoparticles have been investigated due to their suitable characteristics for diverse applications in the fields of biomedicine, electronics, water or wastewater treatment and sensors. Maghemite, magnetite and hematite are the most widely studied iron oxide particles and have ferrimagnetic characteristics. When very small, however, these particles have superparamagnetic properties and are called superparamagnetic iron oxide nanoparticles (SPIONs). Several methods are used for the production of these particles, such as coprecipitation, thermal decomposition and microemulsion. However, the variables of the different types of synthesis must be assessed to achieve greater control over the particles produced. In some studies, it is possible to compare the influence of variations in the factors for production with each of these methods. Thus, researchers use different adaptations of synthesis based on each objective and type of application. With coprecipitation, it is possible to obtain smaller, more uniform particles with adjustments in temperature, pH and the types of reagents used in the process. With thermal decomposition, greater control is needed over the time, temperature and proportion of surfactants and organic and aqueous phases in order to produce smaller particles and a narrower size distribution. With the microemulsion process, the control of the confinement of the micelles formed during synthesis through the proportions of surfactant and oil makes the final particles smaller and less dispersed. These nanoparticles can be used as additives for the creation of new materials, such as magnetic bacterial cellulose, which has different innovative applications. Composites that have SPIONs, which are produced with greater rigour with regards to their size and distribution, have superparamagnetic properties and can be used in medical applications, whereas materials containing larger particles have ferromagnetic applications. To arrive at a particular particle with specific characteristics, researchers must be attentive to both the mechanism selected and the production variables to ensure greater quality and control of the materials produced.

## 1. Introduction

Magnetism is an important property in numerous materials and devices found in daily living, such as electronics. Iron oxides are a widely investigated group of magnetic materials and include materials with specific magnetic properties (ferromagnetic and paramagnetic) that are useful in particular biotechnological applications [1,2].

Iron oxide nanoparticles have a large surface area in relation to their volume, can be used as dopants in the fabrication of composites and generally form magnetic monodomains, which has attracted the attention of a growing number of researchers [3]. Iron oxide nanoparticles have applications in the biomedical field, drug-release systems, the treatment of hyperthermia, magnetic resonance and tissue engineering as well as in sensors, biosensors, water or wastewater treatment and the fabrication of electronic components [4].

The different routes for the obtainment of iron oxide nanoparticles include mechanisms of coprecipitation, thermal decomposition, microemulsion, sol–gel, polyol and hydrothermal synthesis [5]. It is common for authors to state that each specific technique produces larger or smaller particles with a broad or narrow distribution of sizes. However, several studies have shown that these characteristics can be controlled by adjusting the reaction variables, such as the temperature, time, pH, reagents and proportions [6].

Such control is fundamental in certain applications, such as the production of superparamagnetic iron oxide nanoparticles (SPIONs), which are commonly used in the medical field [7,8]. In such applications, the control of the size distribution is essential, as very large particles can interfere with the action of SPIONs. In contrast, nanoparticles can have larger sizes in applications that require materials with ferromagnetic properties, in which the control over the size uniformity is less rigorous [9].

In many applications, iron oxide nanoparticles are incorporated into bacterial cellulose, which is a sustainable, biotechnological compound for the production of novel biotechnological materials that meet the needs of different fields and are used in the fabrication of various devices, such as contrasts for magnetic resonance, sensors and electronics [10].

Several researchers have used methods with specific synthesis protocols to obtain particles for desired applications. Therefore, the aim of the present work was to perform a study on iron oxide nanoparticle formation mechanisms and the effects of the size distribution in different applications, with an emphasis on magnetic bacterial cellulose.

## 2. General Considerations about Magnetism

**Figure 1:**
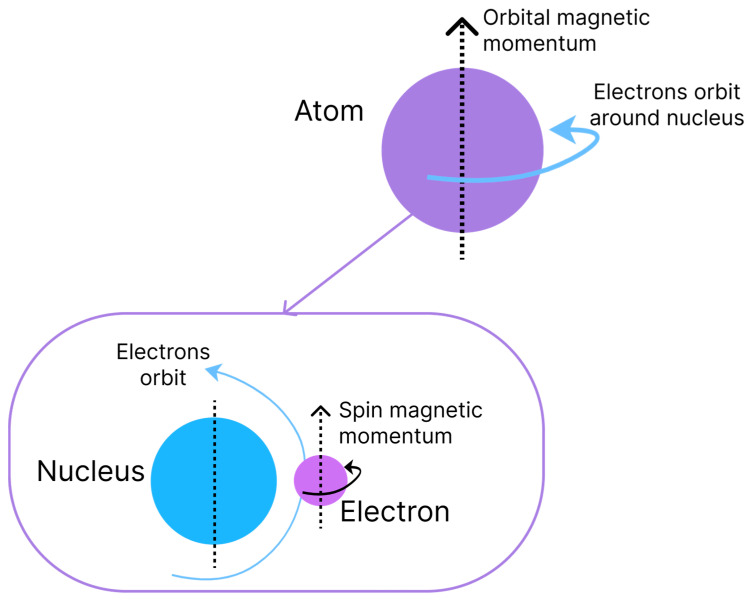
Orbital and rotation movements of electrons respectively generating orbital and spin momentums.

The origin of magnetism is associated with the movements of electrons and the capacity to have an angular moment of the respective spins. Magnetism is an intrinsic characteristic of each material and is due to the interaction and alignment of its electrons when exposed to an external magnetic field, causing properties of attraction or repulsion in other materials [11]. Two types of movement are associated with electrons, which are denominated magnetic moments that are similar to the rotation and orbit of the Earth, as shown in Figure 1. The orbital angular momentum of an electron is given by its movement around the nucleus of an atom, whereas the spin angular momentum is defined by the rotation on its axis [1].

**Figure 2:**
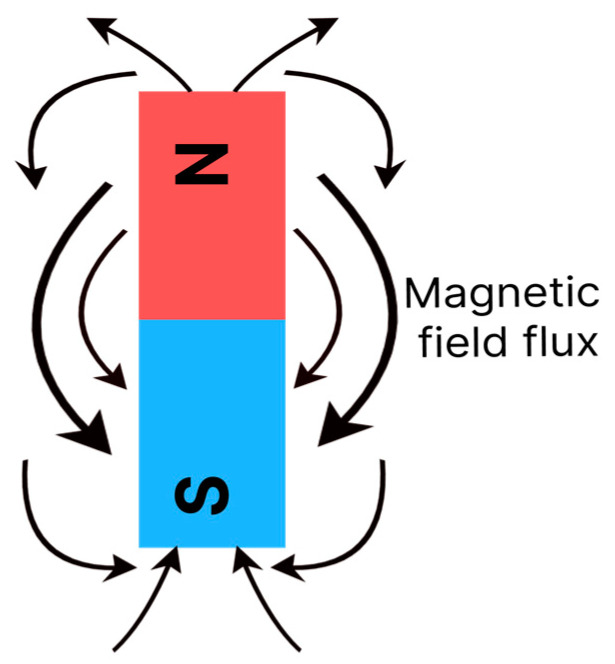
Illustration of magnetic forcefields through flow lines that go from north to magnetic south.

The movement of electrons creates a forcefield in the material, which can be represented by imaginary flow lines with a certain direction and intensity, as shown in Figure 2. The lines are orientated to describe the flow, which goes from magnetic north to south [12]. Some materials magnetise when exposed to an external field or electrical current and others have the capacity to generate fields spontaneously [13].

Materials can behave differently when exposed to an external magnetic field. In response to this field, the electronic spins align in specific manners, generating a resulting magnetic moment. Regions or pieces of material in which all spins are aligned in the same direction are denominated domains [14]. Based on the alignment of these spins and domains, materials are classified as ferromagnetic, paramagnetic, diamagnetic, ferrimagnetic and antiferromagnetic [15].

Diamagnetic: Materials whose atoms do not produce a resulting magnetic moment. This occurs because the magnetic moments in each their atoms cancel each other out. When exposed to an external magnetic field, the spins of diamagnetic materials are positioned against the field [1,11,12,13]. Examples include water, diamonds, graphite and bacterial cellulose.

Paramagnetic: In this case, the spins are arranged in random directions. When exposed to an external magnetic field, however, the spins align in the same direction. With the removal of the external field, the random directions return, as the magnetic interactions among the atoms are too weak to maintain the alignment [1,11,12,13]. Changes in temperature affect the magnitude of the magnetic moments in materials of this type, such as aluminium, magnesium and copper sulphate.

Ferromagnetic: When such materials are submitted to an external magnetic field, the spins—or a large part of the spins—align in the same direction as the field. Due to the strong interaction with each other, the spins continue to be aligned even after the removal of the field [1,11,12,13]. Iron, nickel and cobalt are examples of ferromagnetic materials.

Ferrimagnetic: Similar to ferromagnetic materials, a large part of the spins in these materials aligns in favour of the magnetic external field. Another part aligns contrary to the field, but with a very low intensity. Thus, the resulting movement is different from 0. This alignment remains after the removal of the external field. Magnetite, maghemite and ferrites are examples of ferrimagnetic materials.

Antiferromagnetic: In this classification, the electron spins of the material align in an anti-parallel arrangement when exposed to an external magnetic field [1,11,12,13]. Examples include hematite and nickel oxide.

When a ferromagnetic or ferrimagnetic material is submitted to an external magnetic field, there is a limit at which all spins and domains of the material are aligned in the same direction as the field. This is known as saturation magnetisation (Ms) [1]. After saturation is reached, a further increase in the external field causes no change to the magnetisation of the material. Saturation is used as a parameter to assess the magnetism of a given material with magnetic behaviour. Another characteristic used for assessment is the coercivity field (Hc), which indicates the extent to which a material can withstand an external magnetic field without being demagnetised; that is, the resistance of the material to demagnetisation [12].

Magnetic materials can also be classified as soft or hard depending on the coercivity field. Soft magnetic materials have small coercivity fields and can, therefore, magnetise and demagnetise easily. Such materials can be applied in situations that require instantaneous and variable magnetism, such as in the cores of electromagnets and data registration systems. Hard magnetic materials have permanent magnetisation (greater resistance to demagnetisation), meaning they have a larger coercivity field. Hard magnetic materials are used as permanent magnets [14].

**Figure 3:**
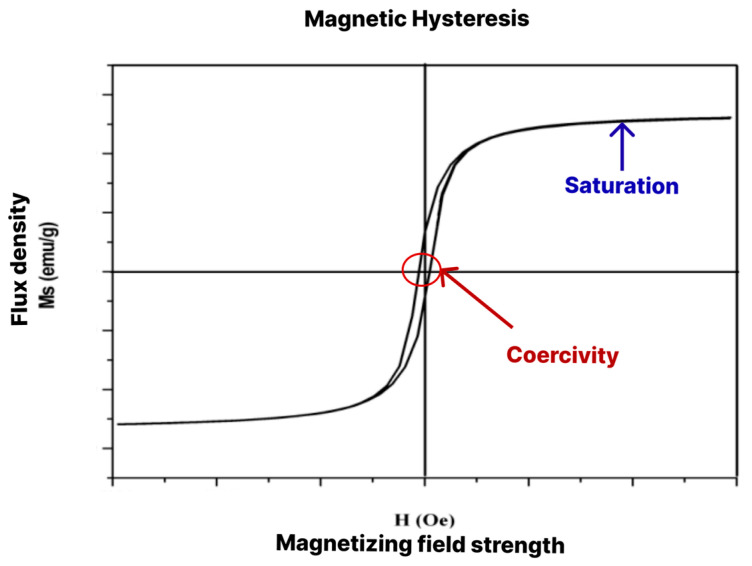
Example of magnetic hysteresis with points of saturation magnetisation and coercivity.

A vibrating sample magnetometer is used to characterise a particular magnetic material and measure its saturation magnetisation (Ms) and coercivity field (H). With this equipment, the material is submitted to a magnetic field until reaching saturation. The field is then gradually inverted to enable the observation of the magnetism remaining in the material [16]. The response generates a hysteresis curve, as exemplified in Figure 3.

### Magnetic Domains in Nanoparticles

Nanometric particles are in a stabilised molecular state; therefore, they have different properties from those with micrometric sizes [6]. Such properties depend on the morphology, crystallinity and size. The main properties are a large surface area in relation to volume, the possibility of being used as additives, the possibility of being coated with other compounds and the formation of magnetic monodomains [17].

**Figure 4:**
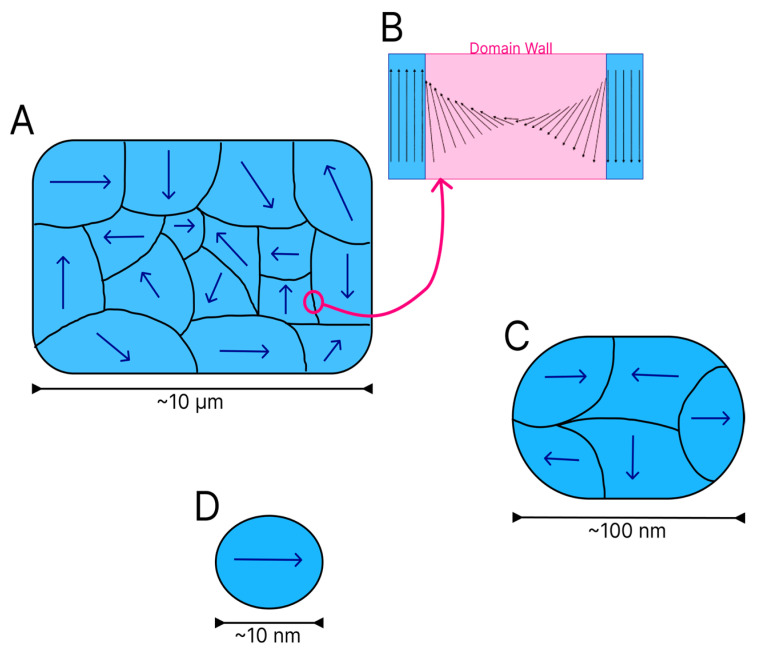
Magnetic domains in particles of different sizes. Multiple domains in materials with micrometric particles (**A**). Highlight of the domain wall (**B**). Fewer multiple domains in material with intermediate dimensions (**C**). Monodomain in particle that reached critical size (**D**).

As mentioned above, the electron spins of a material begin to acquire a specific alignment when in contact with a magnetic field. When a group of spins align in a similar manner, domains are formed [17]. Domains act as small magnets within a ferromagnetic or ferrimagnetic material. Each domain is divided by walls, which are regions with a finite size where there is a transition between adjacent domains. On the domain walls, the magnetic moment vectors spin from one direction to another, as shown in Figure 4B [14].

Several magnetic domains are formed in larger particles (Figure 4A), although the energy cost for the maintenance of the domain wall increases as the dimensions of the material diminish. To arrive at a critical size, the maintenance of a single domain within the material is less costly from an energy standpoint than the reorganisation of the material into multiple domains. The shrinking of particles to a certain size or critical diameter characteristic of each material leads to the formation of magnetic monodomains [17] (Figure 4D). Figure 4 shows a reduction in the quantity of domains in particles of different sizes.

Particles with magnetic monodomains have been widely studied due to their faster, more efficient responses to an external field [17]. In particles of particular materials even smaller than the critical size, magnetisation becomes spontaneous throughout the entire particle, although the direction of magnetisation can change with a thermal fluctuation, indicating a behaviour denominated by superparamagnetism [3]. In this type of behaviour, the system of particles has properties similar to paramagnetism due to the change in moment as a function of temperature, although with greater magnetic moment intensity [2]. The types of nanomaterials most recurrent in the literature that contain magnetic characteristics include the iron oxides magnetite, maghemite and hematite. 

There is also vast research on SPIONs (superparamagnetic iron oxide particles smaller than 20 nm) [3]. Superparamagnetic properties allow nanoparticles, such as SPIONs, to respond in specific ways to alternating high- and low-frequency magnetic fields. Particles of this type do not retain residual magnetism after removal from the external field, thereby preventing agglomerations of particles in specific points. Applications in the medical field, where features like these are desirable, have also demonstrated the use of SPIONs since the 1990s [8].

## 3. Iron Oxides—Generalities and Types

Iron and its derivatives have been used by humans since the end of the Neolithic period. This element is commonly found in the form of oxides and oxyhydroxides, which encompass a large group of materials. The group contains distinct oxides and hydroxides depending on the oxidation state of iron, which can be +2 or +3 [18]. The most common are magnetite, hematite, goethite, maghemite, akaganeite and lepidocrocite.

**Figure 5:**
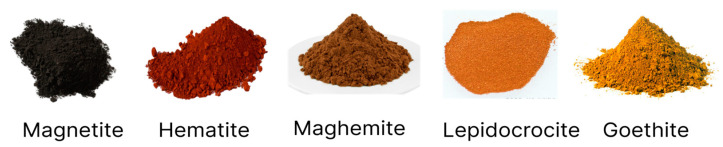
Visual appearance of iron oxides.

The types of iron oxides and oxyhydroxides vary with the quantity of Fe^2+^ and Fe^3+^ ions. Thus, each type acquires different crystalline structures and properties. Figure 5 displays some of these compounds. 

**Figure 6:**
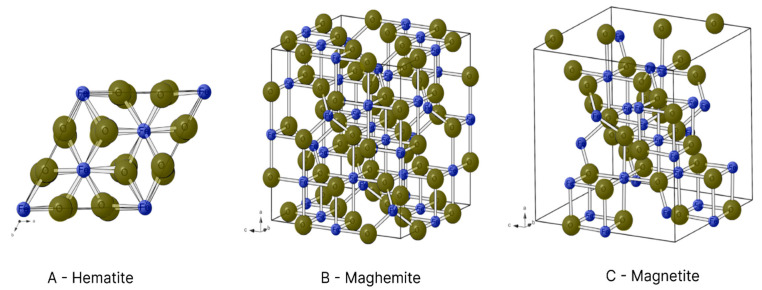
Crystalline structures of hematite (**A**), maghemite (**B**) and magnetite (**C**). Oxygen atoms are shown in yellow and iron atoms in blue.

The magnetic characteristics differ in accordance with the configuration of each iron oxide and are influenced by the quantities of Fe^3+^ and Fe^2+^ in each compound, as well as vacancies in the oxygen atoms within the compounds and the respective crystalline structures [6]. Figure 6 illustrates the crystalline structures of hematite, maghemite and magnetite.

In the hexagonal structure of hematite, each Fe^3+^ ion is surrounded by six O^2−^ ions (Figure 6A). However, each Fe^3+^ ion has a magnetic spin capable of interacting with the spins of neighbouring atoms, forming regions of spontaneous magnetisation (magnetic domains). The interactions among these domains produces the ferromagnetism of hematite [19,20].

Unlike other oxides, magnetite has Fe^3+^ and Fe^2+^ ions in its composition. Fe^3+^ ions with a reverse spinel are found in the octahedral interstices, where they bond to six O^2−^ ions, and tetrahedral interstices, where they bond to four O^2−^ ions. In contrast, Fe^2+^ ions are only found in tetrahedral interstices and bond to four O^2−^ ions (Figure 6C) [19]. The presence of iron ions in different oxidation states and the interactions among their spins give the compound a magnetic moment that is different from 0. Thus, magnetite has ferrimagnetic properties [20]. Another aspect to consider is its critical diameter of 126 nm, as particles smaller than this form magnetic monodomains. Thus, several studies have sought production within this limit [17].

Maghemite is also comprised of Fe^3+^ ions. In its reverse spinel crystalline structure, however, each iron ion is surrounded by six O^2−^ ions in the octahedral interstices and four O^2−^ ions in tetrahedral interstices (Figure 6B) [20]. Its crystalline arrangement is similar to that of magnetite, although cationic vacances are found in its structure due to only having Fe^3+^ ions, which give it a ferrimagnetic behaviour [19]. The critical diameter of maghemite is 50 nm [6].

Industrially, iron oxides and oxyhydroxides are present in the extraction of metallic iron, the production of dyes due to the diversity of colours, the fabrication of magnetic alloys for magnets, etc. Iron oxide nanoparticles are used in different applications, mainly due to their magnetic characteristics, such as electronic devices, inductors, solar cells, water treatment processes, the immobilisation of enzymes, sound amplification equipment, data storage systems, sensors and medical treatment systems (such as hyperthermia, cancer therapy and drug delivery systems) [5,8,9,21,22]. However, the obtainment of iron oxide nanoparticles and SPIONs can be quite challenging, as each of the different obtainment methods has advantages and disadvantages that exert an impact on the characteristics of the end product [7,23].

## 4. Main Iron Oxide Formation Mechanisms 

Several methods are employed to obtain iron oxides with magnetic properties (magnetite, maghemite and hematite), such as physical, chemical and biological routes. Chemical routs are the most widely used mainly due to the greater versatility and variety of methods [2,24]. Each synthesis route confers different properties to the materials, such as the particle size and distribution. Such characteristics have an impact on the final magnetic properties of the material, especially if the goal of the synthesis is the obtainment of nanoparticles [13,17]. 

The iron oxide formation mechanisms described in the literature are coprecipitation, thermal decomposition, microemulsion, the sol–gel method and polyol and hydrothermal syntheses. The most widely used of these methods are coprecipitation, thermal decomposition and microemulsion [2,13,17,23].

### 4.1. Coprecipitation

**Figure 7:**
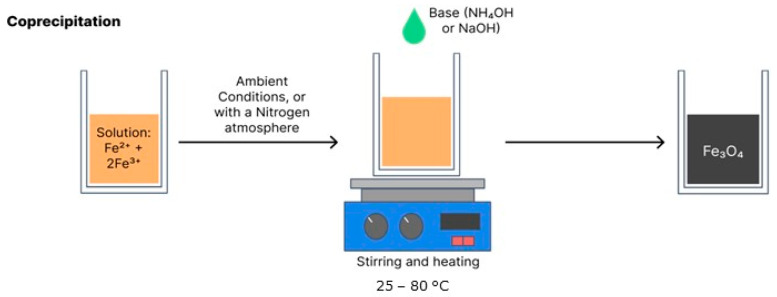
Schematic of magnetite synthesis by coprecipitation.

Coprecipitation is the most widely used synthesis method due to its simple, efficient, low-cost execution and the fact that it can be used on a large scale. Coprecipitation can be performed at room temperature or high temperatures. The synthesis of magnetite and maghemite is performed from ferrous and ferric salts in a certain stoichiometry in an aqueous solution with the addition of a base [16,18]. The final particle sizes and shapes vary depending on the reagents used, such as nitrates, chlorides and sulphates. Equation (1) shows the general reaction for the obtainment of Fe_3_O_4_ and Figure 7 illustrates coprecipitation synthesis [16].

Fe^2+^ + 2Fe^3+^ + 8OH^−^ → Fe_3_O_4_ + 4H_2_O (1)

**Figure 8:**
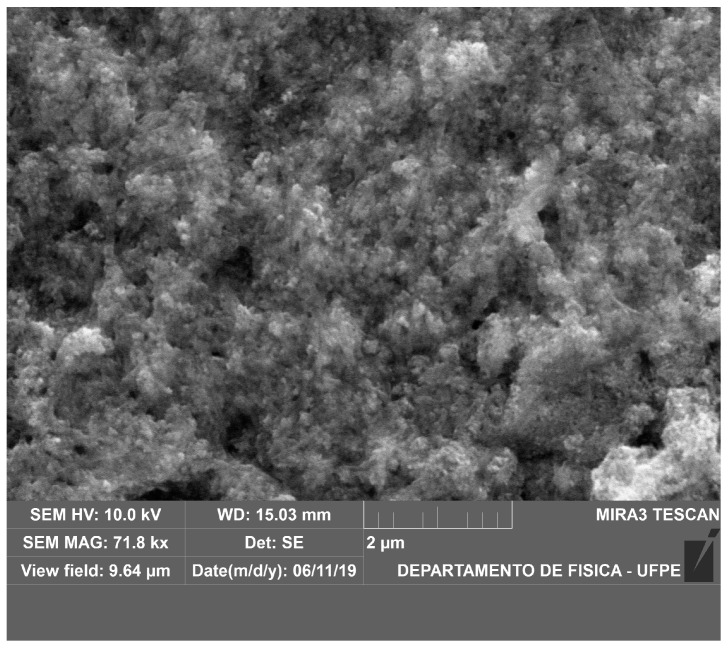
SEM image of Fe_3_O_4_ obtained by coprecipitation inside bacterial cellulose film.

Figure 8 shows an SEM image of magnetite particles synthesised by the authors’ research group through coprecipitation synthesis. The particles were produced through a solution with iron II and III chloride and NH_3_OH as an oxidative agent at room temperature. The synthesis was carried out inside a bacterial cellulose film.

Yazdani and Seddigh [25] and Chanthiwong et al. [26] conducted comparative studies of coprecipitation methods for the obtainment of magnetite using different combinations of iron salts. These combinations are listed in Table 1.

Yazdani and Seddigh [25] found that Fe_3_O_4_ crystallites synthesised by the G1 route with iron chlorides obtained an average size of 10.03 nm, whereas those synthesised by the G5 route had a smaller average size (5.10 nm). The G1 route also had particles with a broader size distribution. In terms of morphology, all routes produced Fe_3_O_4_ nanoparticles with semi-spherical shapes. The particles synthesised using the G1 route had the highest saturation magnetisation (53.38 emu/g) and those synthesised using the G5 had the lowest (30.50 emu/g).

Chanthiwong et al. [26] obtained Fe_3_O_4_ crystallites via the (C + C) route that had a mean size of 12.2 ± 1.8 nm. Larger crystallites were synthesised using the (A + C) route (13.8 ± 2.1 nm). The magnetic saturation of the particles obtained by the (C + C) route was 61.5 emu/g (highest saturation described in the study). Yazdani and Seddigh in 2016 reported similar findings. Table 2 offers greater detail on the results described by the authors.

According to Yazdani and Seddigh [25], the differences in size among the routes may be explained by the control exercised by the ionic strength of the reagents over the growth of the oxide particles in an aqueous solution, as lower ionic strength results in a smaller size of the synthesised particles. The ionic strength of the reagents in the G1 route was the weakest, whereas that of the reagents in the G5 route was the strongest. 

Another factor in the choice of reagents that Yazdani and Seddigh [25] highlight as exerting an influence on the final particle size is the electric double layer theory, as some routes with the same ionic strength (G1 and G3, G4 and G6) produced particles of different sizes. According to this theory, two layers are formed when a charged surface is exposed to a fluid. The first is composed of ions that are attracted to the surface and the second is composed of ions dispersed in the fluid that are attracted by the first layer. The first layer has a stronger attraction to the surface and is charged with cations and anions, whereas a combination of cations and anions in a state of equilibrium is found in the second layer [25,27]. 

When the surface is negatively charged, the first layer is filled with cations and there is a change in the anions in the second layer. This change has an effect on the thickness of the electrostatic layer that envelopes the particle, with larger anions leading to a greater layer thickness. Thus, an increase occurs in diffusion resistance and the dimensions of the final crystals diminish [25,27]. In the study by Yazdani and Seddigh [25], the sizes of the anions in the precursor reagents were 127 pm (Cl^−^), 258 pm (SO_4_^−^) and 179 pm (NO_3_^−^). The smallest particles were synthesised with SO_4_^−^ and the largest were produced with Cl^−^, which is in line with the double layer theory.

The pH and temperature at which the reaction occurs are other factors that can affect the particle size with this method. In a magnetite coprecipitation reaction, the precipitate of the nanoparticles is formed at pH values higher than 8 due to the greater concentration of anions in the medium, which is linked to the double layer theory [4]. A high pH also leads to a broader distribution of particle sizes due to the reduction in the surface tension of the particles [2]. Riaz et al. [28] performed synthesis via coprecipitation with changes in pH (2, 4, 6, 8 and 10) and found particle sizes between 25 and 30 nm (very narrow distribution) at pHs 2, 4 and 6, whereas the size distribution was from 50 to 100 nm at pH 8.

The reaction temperature can also exert an influence on the distribution of particle sizes at the end of the process. Studies state that reactions in which the temperatures ranged from 25 °C (room temperature) to 45 °C produced smaller crystallites, whereas the size increased at higher initial and final temperatures. High temperatures favour the greater growth of magnetite crystallites, as heat furnishes more energy to the particles and favours more collisions between particles [28]. Another important factor is the presence of oxygen in the process. The agitation of the system causes oxygenation, which can lead to the oxidation of magnetite and maghemite. Thus, many researchers pump in nitrogen or argonium to create an oxygen-free atmosphere [6,16].

The influence of the choice of variables, such as reagents, pH, temperature and oxygenation, on the nanoparticle production process via coprecipitation is evident. When using this route, one must determine whether larger or smaller particles with greater or less saturation are desired in order to select the variables to be used in the process. 

### 4.2. Thermal Decomposition

According to the literature, thermal composition is the method that produces particles of smaller sizes. Organometallic compounds (such as iron acetylacetonate, iron oleate and carbonyl iron) are decomposed at high temperatures, dissolved in organic solvents with a high boiling point (e.g., phenyl or diphenyl ether) and stabilisers or surfactants (e.g., fatty acids and fatty amines) [6,29]. The reaction occurs in an aqueous medium in a reactor or autoclave. Variables such as the types of reagents, proportion of solvents, temperature and reaction time should be monitored to obtain greater control over the morphology, size and crystallinity of the resulting nanoparticles [2,5].

**Figure 9:**
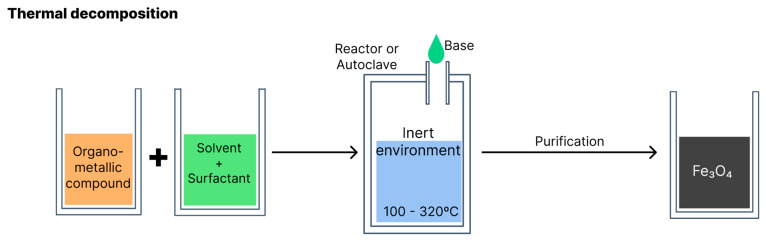
Schematic of magnetite production via thermal decomposition.

Figure 9 illustrates synthesis via thermal decomposition.

Koo et al. [30] created a table with some variables that should be controlled during synthesis by thermal decomposition for the formation of magnetite and to influence the size, distribution and magnetism of the final particles. Some of this information is listed in Table 3. The authors presented three production variables (combination of solvents + surfactant, time and temperature) and the influence exerted by the variance in these factors. Increases in temperature (220, 265, 300 and 330 °C) led to increases in the final particle size (3, 5, 9 and 24 nm) and size distribution. According to the authors, these effects occurred due to the disorderly growth of the crystals caused by the increase in temperature. The reaction time was another factor that contributed to these increases, which may be explained by the Ostwald ripening phenomenon, by which suspended smaller crystals within a stage continue to dissolve, whereas larger crystals continue to grow [30]. 

Estévez et al. [31] found a result that complements the relationship between the temperature and particle size described by Koo et al. [30]. The authors synthesised magnetite nanoparticles using iron oleate as the precursor, oleic acid as the surfactant and octadecene as the solvent. The reaction occurred at 315 °C for one hour. The resulting particles had an octahedral shape and a mean size of 11 ± 2.6 nm. This size obtained at a temperature of 315 °C is within the range displayed in Table 3, in which nanoparticles were obtained at sizes of 9 nm at 300 °C and 24 nm at 330 °C. The magnetic saturation levels of the nanoparticles ranged from 0.71 emu/g (at a temperature of 5 K) to 0.53 emu/g (at a temperature of 290 K). The change in magnetic saturation with the change in temperature demonstrated the superparamagnetic characteristics of the particles produced.

However, syntheses with higher temperatures and longer reaction times produce crystals with greater magnetisation. Therefore, researchers seek a better size distribution through the choice of solvents and surfactants. Table 3 displays information on how synthesis in the absence of solvents (reaction medium with surfactants alone) can contribute to better control over the distribution of particle sizes despite high temperatures and long reaction times. 

In this synthesis method, Ostwald ripening did not occur and particles with greater magnetisation were produced. This effect occurs because the stabilisers and surfactants are able to decelerate the nucleation process of the crystal, thereby affecting the adsorption of additional particles, restricting nanocrystal growth and favouring the production of smaller particles [29,30].

The table shows that the temperature, time and proportion of solvents exert a considerable influence on the final particle size and distribution.

### 4.3. Microemulsion

**Figure 10:**
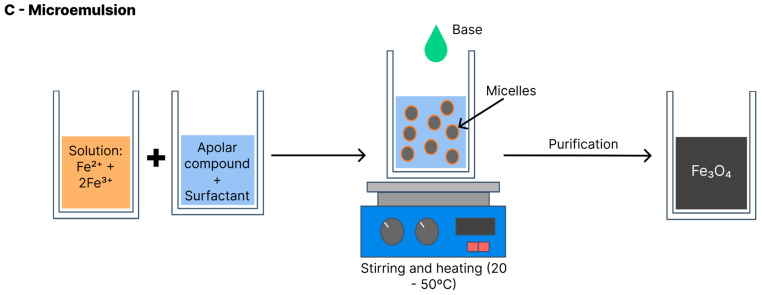
Schematic of magnetite synthesis via microemulsion.

Also known as the micellar method (Figure 10), the iron oxide formation reaction with this method is performed at a biphasic interface (generally water and oil). In most cases, the reagents are dissolved in the aqueous phase and a surfactant is dissolved in the organic phase [29]. All components are vigorously shaken and the collision between molecules leads to the formation of micelles with the interposition of the surfactant, which serve as nanoreactors for the iron oxide formation. The nanoparticles precipitate within the micelles. Thus, the particle size and distribution depend on the size of the micelles. Variables such as the agitation velocity, temperature, type of surfactant and proportion of solvents influence the micelle formation and the final size of the nanoparticles [6]. This method can be performed with heat (20–50 °C) or at room temperature [5].

Okoli et al. [32] studied the influence of the water/oil ratio in this method. The authors performed syntheses through two pathways. The first was oil/water (o/w) comprised of 21.4% in mass of a non-ionic surfactant, 14% of the organic phase (composed of a solution of hexane and iron III 2- ethyl hexanoate at a proportion of 2% iron in the solution) and 64.5% of the aqueous phase. The other pathway was water/oil (w/o) using a cationic surfactant (1-butanol), an organic phase composed of n-octane and an aqueous containing the salts FeCl_3_ and FeCl_2_ at a 2:1 molar proportion. The proportions of these components were respectively 0.6:0.5:3:1.3 in a molar fraction. The precipitating agent in both pathways was a 30% NH_4_OH (volume) solution, to which the systems were added until the pH reached 11. The reactions occurred at a temperature of 30 °C for 48 h. The nanoparticles were purified through centrifugation and washing with water and ethanol. 

At the end of the process, the authors obtained maghemite nanoparticles. Those produced in w/o had an elongated shape, with widths ranging from 5 to 10 nm and lengths from 20 to 50 nm, coexisting with globular particles with a diameter range of 5 to 8 nm. The authors considered the average particle size to be 9.2 nm with this route, whereas particles with a globular shape in the range of 2–3 nm were produced with the o/w route.

With regards to magnetism, the saturation magnetisation rates were 30 emu/g for the compounds synthesised with the w/o route and 10 emu/g for those synthesised with the o/w route. According to the authors, the reaction in the o/w occurred more quickly, prioritising nucleation over growth, leading to smaller particles. Another possibility raised was the coating of the nanoparticles with the iron III 2- ethyl hexanoate precursor, which inhibited greater growth of the maghemite particles [32]. 

Darbandi et al. [33] also studied the influence of the proportions of water, oil and surfactant on the size and distribution of maghemite and magnetite particles. For the synthesis, the authors used an aqueous solution with FeCl_2_ and FeCl_3_ at a ratio of 1:2 and a surfactant denominated IGEPAL^®^ CO-520 (polyoxyethylene (5) nonylphenyl ether) diluted in cyclohexane. The synthesis occurred at room temperature with a nitrogen flow. The oxidant agent was a 33% (by volume) solution of NH_4_OH. The proportions described in the article followed the (cyclohexane + water)/surfactant ratios. The ratios calculated were 4.1 (reaction with most surfactant), 10.5 and 16.2 (reaction with least surfactant). The respective sizes of the particles produced in the proportions of water and oil listed were 3.2 ± 0.5 nm, 6.3 ± 0.9 nm and 9.1 ± 1.4 nm. The final nanoparticle size was smaller and the distribution was narrower using the route with the largest quantity of surfactant, making the final result more uniform.

In the study by Okoli et al. [32], the particle growth was limited due to the barrier formed around the micelle. In the study by Darbandi et al. [33], however, isolation was reinforced due to the greater quantity of surfactant. Based on these studies, the isolation of the micelle is a crucial factor for the formation of iron oxide particles in its interior. Factors such as the proportion of surfactants and reactions in a w/o or o/w medium, which assist in the confinement of the micelles, also lead to smaller, more uniform nanoparticles.

### 4.4. Comparison of Coprecipitation, Thermal Decomposition and Microemulsion Methods

As discussed above, all nanoparticle formation mechanisms cited depend on variables such as the temperature, time, selection of reagents and proportions in order to have effective control over the formation of particles. Comparative studies of the variables of the processes demonstrate differences in the dimensions of the particles obtained as well as their magnetisation and size distribution. Thus, it is possible to produce iron oxides with different characteristics not only using different synthesis methods but also with variations in the variables of each method. 

Yusoff et al. [6] created a comparative table with some practical aspects of each method, which are displayed in Table 4.

The selection of a method that provides the desired properties of iron oxide nanoparticles for the objective largely depends on the research group. As discussed above, it is possible to obtain iron oxide nanoparticles with similar characteristics, such as size magnetisation, with the use of different methods under certain process conditions [6].

Coprecipitation is the most widely adopted method in studies due to its simplicity. However, this method produces somewhat larger nanoparticles and adjustments must be made in pH and temperature to obtain greater uniformity [24]. If the objective is to obtain particles with greater control over their morphology and uniformity (narrower size distribution), the thermal decomposition mechanism is more advantageous. However, this process requires more time, higher temperatures and more specific apparatuses, making it more complex [29]. Microemulsion ensures high control over the morphology and size distribution, and like coprecipitation is a faster method involving lower temperatures and normal environmental conditions. However, this process has low yields and high consumption rates of solvents, which makes scaling difficult [4].

Thus, when selecting a method, researchers should consider their objectives and priorities and choose that which best suits each work.

## 5. Main Effects of Size Distribution in Different Applications 

Among the applications and studies aimed at the synthesis of iron oxide nanoparticles, researchers generally seek materials that result in the formation of monodomains. The particle size exerts a considerable influence on the magnetic properties, as seen in the studies cited above. In some applications, however, there is greater concern with the size distribution and greater uniformity is desirable, especially if the material of interest is a SPION [7,24].

In applications that do not require greater rigour in the control of the size distribution, the objectives are to produce particles with ferrimagnetic characteristics and high saturation magnetisation or like Fenton’s reagent. Jędrzak et al. [34] synthesised magnetite nanoparticles via coprecipitation for the production of a blood glucose sensor and demonstrated efficiency in the quantification of the compound. The particles had an average size range of 8 to 12 nm, which was satisfactory for the application. The use of magnetic particles as transducers is common in sensors. In the case of blood glucose biosensors, however, magnetite can be used as a synthetic enzyme and as a Fenton reagent due to the presence of Fe^2+^, which can mimic the action of the enzyme peroxidase (which is present in the reactive strips of the sensor) [9]. 

In another study in which the authors obtained a broad distribution, Hwang et al. [35] produced a sensor for detecting phenylhydrazine. Iron oxide nanoparticles were synthesised and used to fabricate an electrode. The nanoparticles had a mean size of 40 ± 10 nm, with a somewhat large variation range that was acceptable for the application. Xu et al. [36] conducted a literature review to investigate the application of iron nanoparticles in supercapacitators and reported several studies in which the particle size distribution was broad but did not exert a negative impact on the applications.

In the case of SPIONs, the size distribution is crucial, as one larger particle synthesised with others could surpass the diameter limit and not have the desired superparamagnetic characteristics. Such characteristics in SPIONs are important in biomedical applications, as the nanoparticles are guided to the location of interest by the application of an external magnetic field to be used as necrophages or to increase the temperature around cancer cells to eliminate them [16].

Nanoparticles larger than SPIONs have ferrimagnetic behaviour, which hampers the action of the superparamagnetic nanoparticles. For instance, if the synthesised nanoparticles do not have uniform sizes, such as a mixture of SPIONs and larger particles, all particles will magnetise with the application of a magnetic field, although the SPIONs will return to their original state with the removal of the field, whereas the larger particles will maintain their magnetic alignment [3,37]. Taking this into consideration in a hypothetical situation, the action of a drug delivery system that has iron oxide particles of various sizes will be compromised, as the larger particles would maintain their magnetic action even after the removal of the magnetic field that guided them and would cause the clustering of SPIONs around them, leading to imprecision in the system [5,7].

## 6. Application in Bacterial Cellulose and Effect of Size Distribution

**Figure 11:**
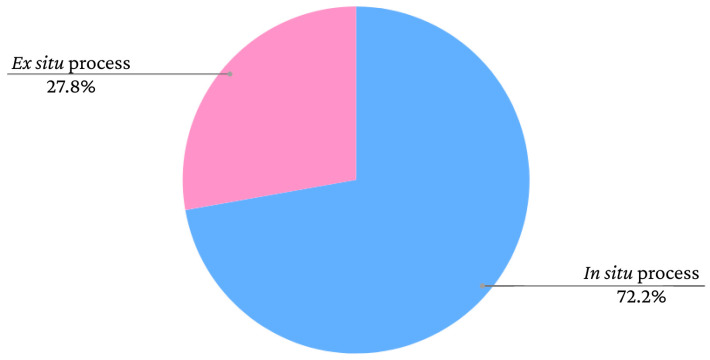
Frequency rates of iron oxide incorporation methods in bacterial cellulose in studies found in the literature.

Iron oxide nanoparticles can be used as dopants to produce novel composites and magnetic materials. The matrices of these materials could be polymers, for example [38,39,40]. Bacterial cellulose (BC) is a biomaterial with sustainable production through the fermentation of bacteria that has unique properties, such as biocompatibility, biodegradability and high water absorption capacity [38,39,40,41]. The incorporation of iron oxide nanoparticles in BC can occur in situ, when the production process of the additives is performed within the BC, or ex situ, when the iron oxide is synthesised separately and subsequently inserted into the BC [10]. The graph presented in Figure 11 shows the frequency rates of iron oxide incorporation processes in bacterial cellulose films according to data extracted from the study by Souza et al. [10].

As seen in the graph, there is a greater tendency to adopt in situ methods. However, the nanoparticles adhere to the BC fibres similarly in both cases. According to Mira-Cuenca et al. [40], iron nanoparticles adhere to BC fibres through hydrogen bridges. The fibres have nanometric thicknesses, which facilitates the adherence of the nanoparticles. Marins et al. [42] and Salidkul et al. [43] used coprecipitation to obtain iron oxide particles, although used different incorporation methods. Marins et al. [42] performed coprecipitation in situ, whereas Salidkul et al. [43] performed the method ex situ. However, the formed nanoparticles had a similar arrangement in the BC films, adhering throughout the fibres in a uniform manner.

As mentioned above, the applications vary depending on characteristics such as the particle size and distribution. In the studies presented above, the authors suggest distinct applications for BC composites with iron oxide. In the study by Salidkul et al. [43], the particles obtained a mean size of 63.2 ± 6.2 nm and the authors suggested applications in the fields of magnetic shielding, data storage and electromagnetic absorption. Marins et al. (2013) synthesised iron oxide with different variations in time. In the two subsamples obtained, the nanoparticles had mean sizes of 10 ± 1 to 13.4 ± 1 (smaller size and narrower distribution). Thus, the authors also suggested medical applications for the material obtained. 

The study by Mira-Cuenca [40] exemplifies the application of a material composed of BC and iron oxide that required greater control in the size distribution of the final particles. The researchers developed a device for the contrast agent in magnetic resonance with SPIONs synthesised through in situ thermal decomposition in triturated BC membranes. Five grams of triturated BC was mixed into 40 mL of benzyl alcohol with 1100 mg of the precursor tris(acetylacetonate)iron (III) (Fe(acac)3). The mixture was first heated at 60 °C for 5 min and then at 210 °C for 10 min in a microwave oven. The mean SPION size was 13 ± 5 nm. The material was used a magnetic paint and was deposited on dry BC films using the screen-printing technique. The authors tested the device in pieces of pork loin in a magnetic resonance machine with a T2 relaxation time (spin-spin) and the device proved to be a good contrast agent. As a result, the authors suggested the application of the material in internal bone implants, enabling better monitoring of the implants through magnetic resonance images and avoiding the need for additional surgeries.

In this application, the control of the size distribution is fundamental, as superparamagnetic particles have a longer relaxation time, which is an important factor in contrasts for magnetic resonance.

As demonstrated in some articles, there are various applications for iron oxide nanoparticles. However, each requires a larger or smaller variations in size distribution and specific characteristics. Thus, BC-derived materials with the addition of iron oxide nanoparticles undergo the same selectivity depending on the additive material and can have applications in different fields.

## 7. Conclusions

It is possible to obtain iron oxide nanoparticles with different characteristics depending on the variables adopted during the execution of each of the production methods. The temperature, pH, reagents and specific proportions can provide greater uniformity in the size distribution of the particles. 

The particles must be smaller in biomedical applications and SPIONs, for which greater rigour is required in the distribution of sizes. With the coprecipitation method, such control can be achieved with adjustments in pH and temperature to lower levels and via the choice of reagents, such as FeSO_4_.7H_2_O, Fe(NO_3_)_3_.9H_2_O, Fe(C_2_H_3_O_2_)_2_ and 2 Fe(NO_3_)_3_. With the thermal decomposition method, this control can be achieved through shorter reaction times and lower reaction temperatures as well as the use of surfactants. With the microemulsion method, the smaller sizes and better distribution require better isolation of the micelles formed; the larger proportions of surfactant and the organic phase in the medium assist in this purpose. 

For applications that require ferromagnetic characteristics, other factors can be used to obtain smaller particles, such as the use of iron chloride in coprecipitation methods, longer reaction times and higher temperatures in the thermal decomposition process and smaller quantities of surfactants and organic compounds with the microemulsion mechanism.

It is possible to incorporate iron oxide nanoparticles into bacterial cellulose materials in situ and ex situ, thereby obtaining biotechnological materials with unique properties and promising applications. The properties of the incorporated nanoparticles will define the possible applications of the material.

Materials derived from bacterial cellulose supplemented with iron oxides have great biotechnological potential for various types of applications. Applications in the medical and electronics areas have a great demand for biocompatible and sustainable magnetic materials, composites made from bacterial cellulose and iron oxides, as their versatility makes them an innovative solution to meet this demand.

## Figures and Tables

**Table 1:** Combinations of reagents described in articles by Yazdani and Seddigh [25] and Chanthiwong et al. [26].

Article by Yazdani and Seddigh [24]		Article by Chanthiwong et al. [25]	
Name of route	Corresponding route	Name of route	Corresponding route
G1	FeCl_2_.4H_2_O + FeCl_3_.6H_2_O	C + C	FeCl_2_ + 2FeCl_3_
G2	FeCl_2_.4H_2_O + Fe_2_(SO_4_)_3_	S + C	FeSO_4_ + 2FeCl_3_
G3	FeCl_2_.4H_2_O + Fe(NO_3_)_3_.9H_2_O	A + C	Fe (C_2_H_3_O_2_)_2_ + FeCl_3_
G4	FeSO_4_.7H_2_O + FeCl_3_.6H_2_O	C + N	FeCl_2_ + 2Fe(NO_3_)_3_
G5	FeSO_4_.7H_2_O + Fe_2_(SO_4_)_3_	S + N	FeSO_4_ + 2Fe (NO_3_)_3_
G6	FeSO_4_.7H_2_O + Fe(NO_3_)_3_.9H_2_O	A + N	Fe(C_2_H_3_O_2_)_2_ + 2 Fe(NO_3_)_3_

**Table 2:** Results found by Yazdani and Seddigh [25] for the synthesis of Fe_3_O_4_ nanoparticles with different reagents.

Study	Name of Routes	Corresponding Synthesis Routes	Size of Crystallite (XRD) (nm)	Size of Crystallite (TEM) (nm)	Saturation Magnetisation (emu/g)
Yazdani and Seddigh [24]	G1	FeCl_2_.4H_2_O + FeCl_3_.6H_2_O	10.03	11.92	53.38
	G2	FeCl_2_.4H_2_O + Fe_2_(SO_4_)_3_	6.60	-	35.10
	G3	FeCl_2_.4H_2_O + Fe(NO_3_)_3_.9H_2_O	8.86	-	51.50
	G4	FeSO_4_.7H_2_O + FeCl_3_.6H_2_O	8.70	-	51.20
	G5	FeSO_4_.7H_2_O + Fe_2_(SO_4_)_3_	5.10	5.12	30.50
	G6	FeSO_4_.7H_2_O + Fe(NO_3_)_3_.9H_2_O	8.23	-	43.5
Chanthiwong et al. [25]	(C + C)	FeCl_2_ + 2FeCl_3_	12.2 ± 1.8	11.3 ± 2.0	61.5 ± 3.1
	(S + C)	FeSO_4_ + 2FeCl_3_	12.4 ± 1.9	11.1 ± 3.0	54.5 ± 2.7
	(A + C)	Fe (C_2_H_3_O_2_)_2_ + FeCl_3_	13.8 ± 2.1	11.3 ± 2.7	60.6 ± 3.0
	(C + N)	FeCl_2_ + 2Fe(NO_3_)_3_	12.4 ± 1.9	10.2 ± 2.0	56.3 ± 2.8
	(S + N)	FeSO_4_ + 2Fe(NO_3_)_3_	11.2 ± 1.7	10.9 ± 2.0	56.6 ± 2.8
	(A + N)	Fe(C_2_H_3_O_2_)_2_ + 2 Fe(NO_3_)_3_	10.4 ± 1.6	9.4 ± 2.2	57.5 ± 2.9

**Table 3:** Magnetite synthesis variables via thermal decomposition.

Variables of Influence	Solvent + Surfactant	Temp. (°C)	Time (h)	Particle Size (nm)	Saturation Magnetisation (emu/g)	Size Distribution
Reaction temperature	BET + OM	220	2	3	46	Small
	PET + OM	265		5	51	Small
	BET + OM	300		9	60	Relatively large
	ODE + OM	330		24	74	Large
Reaction time and surfactant	BET + OM	300	0.5	7	57	Relatively small
			4	12	65	Very large
	BET + OM + OA		0.5	6	-	Very small
			4	14	67	Very small
Absence of solvent	OM	300	0.5	8	-	Small
			4	10	58	Relatively small
	OM + OA		0.5	5	-	Muito Small
			2	6	58	Small
			24	11	71	Small
		330	0.5	7	-	Relatively small
			4	10	76	Relatively small

OM—oleylamine; OA—oleic acid; BET—dibenzyl ether; PET—diphenyl ether; ODE—1-octadecene.

**Table 4:** Practical aspects of coprecipitation, thermal decomposition and microemulsion synthesis methods for the obtainment of iron oxide nanoparticles.

Method	Difficulty	Conditions of Environment	Reaction Temperature (°C)	Reaction Time	Size Distribuion	Control of Shape	Yield
Coprecipitation	Simple	With or without oxygen-fee atmosphere	20–90	Minutes or hours	Relatively restricted	Medium	Very high
Theramal decomposition	Complex	Inert environment	100–320	Hours or days	Very restricted	Very high	Very high
Microemulsion	Complex	Normal conditions	20–50	Hours	Relatively restricted	High	Low

## Data Availability

Not applicable.
